# A Scatter-and-Gather Spiking Convolutional Neural Network on a Reconfigurable Neuromorphic Hardware

**DOI:** 10.3389/fnins.2021.694170

**Published:** 2021-11-16

**Authors:** Chenglong Zou, Xiaoxin Cui, Yisong Kuang, Kefei Liu, Yuan Wang, Xinan Wang, Ru Huang

**Affiliations:** ^1^Institute of Microelectronics, Peking University, Beijing, China; ^2^School of ECE, Peking University Shenzhen Graduate School, Shenzhen, China

**Keywords:** convolutional neural network, spiking neural network, network quantization, network conversion, neuromorphic hardware, network mapping

## Abstract

Artificial neural networks (ANNs), like convolutional neural networks (CNNs), have achieved the state-of-the-art results for many machine learning tasks. However, inference with large-scale full-precision CNNs must cause substantial energy consumption and memory occupation, which seriously hinders their deployment on mobile and embedded systems. Highly inspired from biological brain, spiking neural networks (SNNs) are emerging as new solutions because of natural superiority in brain-like learning and great energy efficiency with event-driven communication and computation. Nevertheless, training a deep SNN remains a main challenge and there is usually a big accuracy gap between ANNs and SNNs. In this paper, we introduce a hardware-friendly conversion algorithm called “scatter-and-gather” to convert quantized ANNs to lossless SNNs, where neurons are connected with ternary {−1,0,1} synaptic weights. Each spiking neuron is stateless and more like original McCulloch and Pitts model, because it fires at most one spike and need be reset at each time step. Furthermore, we develop an incremental mapping framework to demonstrate efficient network deployments on a reconfigurable neuromorphic chip. Experimental results show our spiking LeNet on MNIST and VGG-Net on CIFAR-10 datasetobtain 99.37% and 91.91% classification accuracy, respectively. Besides, the presented mapping algorithm manages network deployment on our neuromorphic chip with maximum resource efficiency and excellent flexibility. Our four-spike LeNet and VGG-Net on chip can achieve respective real-time inference speed of 0.38 ms/image, 3.24 ms/image, and an average power consumption of 0.28 mJ/image and 2.3 mJ/image at 0.9 V, 252 MHz, which is nearly two orders of magnitude more efficient than traditional GPUs.

## 1. Introduction

Deep convolutional neural network (CNN) architectures such as VGG-Net (Simonyan and Zisserman, 2014) and ResNet (He et al., 2016) have achieved close to, even beyond human-level performance in many computer vision tasks such as image classification (Russakovsky et al., 2015) and object detection (Lin et al., 2014) in recent years. However, these large-scale models usually consist of tens of millions of parameters, and compute with massive high-precision (32/64 bits) fixed-point or floating-point multiply-accumulation (MAC) operations. Although network training can be implemented on a cloud server equipped with powerful CPUs or GPUs using backpropagation algorithm (Rumelhart et al., 1986), inference at edge still inevitably requires vast power and memory budget. Lots of works presented various compression (Deng et al., 2020) and quantization methods (Hubara et al., 2016) of neural network or concentrated on less memory access and pipeline optimizing in custom CNN accelerators (Lecun, 2019; Chen et al., 2020), which greatly improved computation efficiency and reduced power consumption.

Considering another kind of emerging approach to incorporate biological plausibility of brain-inspired models and efficient neuromorphic hardware primitives, spiking neural networks (SNNs) (Grning and Bohte, 2014) attract more attention. SNNs inherently communicate and compute with one-bit spike signals and low-precision synapses toward an event-driven information processing paradigm (consuming energy only when necessary) (Sheik et al., 2013; Deng et al., 2020). It has been proved that SNNs are very suitable to be implemented on large-scale distributed neuromorphic chip with impressive energy efficiency (Cassidy et al., 2013; Schuman et al., 2017). For example, a single TrueNorth chip (Akopyan et al., 2015) supports real-time running of 1 million neurons and 256 million synapses with only 70 mW power consumption. Tianjic chip (Deng et al., 2020) is composed of 156 functional neuromorphic core, and achieve several orders of magnitude of energy efficiency compared with common platforms like CPUs or GPUs.

However, training a high-accuracy SNN remains a main challenge due to discrete spike representation and non-differentiable threshold function (Tavanaei et al., 2018). To date, various methods have been applied to construct SNNs with comparable accuracy to conventional CNNs. Some works adopt bioinspired learning rules like unsupervised spike-timing dependent plasticity (STDP) (Falez et al., 2019; Lobov et al., 2020) for feature extraction. However, these layer-by-layer training algorithms usually perform less efficiently in deep architectures. For supervised learning like SpikeProp (Bohtea et al., 2002) and Tempotron (Gutig and Sompolinsky, 2006), they also fail to deal with practical tasks like CIFAR-10 (Krizhevsky and Hinton, 2009) classification. Recent works (Lee et al., 2016, 2020; Wu et al., 2018; Wei et al., 2020; Yang et al., 2021a) use different pseudo-derivative methods (also called surrogate gradient) to define the derivative of the threshold-triggered working mechanism. Thus, the SNNs could be optimized with gradient descent algorithms as artificial neural networks (ANNs) and achieve good accuracies with fast response speed, but a unified and effective surrogate function is the key problem for these methods.

ANN-to-SNN conversion is another popular solution, which tries to match firing rates of spiking neurons and analog activations of ANNs. Esser et al. (2016) presented a simple BNN-to-SNN conversion algorithm, where spike signals are coded within only one time step, so each neuron will fire at most once. Binary SNNs can achieve a great model compression rate with the least resource and power budgets and fastest inference speed with an acceptable loss of accuracy on MNIST (Lecun and Bottou, 1998) and CIFAR-10 (Krizhevsky and Hinton, 2009) datasets. A more common approach is to map the parameters of a ReLU-based ANN to that of an equivalent SNN. Studies (Bodo et al., 2017; Xu et al., 2017) have found that SNNs can be converted from trained high-accuracy CNNs efficiently by the means of data-based threshold or weight normalization. However, the network performances depend on empirical statistics of average firing rate, and require dozens even hundreds of time steps to get a stable accuracy. This may give a large energy and latency budget for hardware implementation. Besides, the final accuracy is still declining when compared with its ANN counterpart due to accumulated errors of spike approximation in higher layers (Bodo et al., 2017; Rueckauer and Liu, 2018; Yousefzadeh et al., 2019).

This work aims to overcome the aforementioned drawbacks in ANN-to-SNN conversion process and hardware implementation, i.e., to present a more accurate, general, and hardware-friendly conversion method, which is compatible with contemporary neuromorphic hardware. For this purpose, we first introduce an adjustable quantized algorithm in ANN training to minimize the spike approximation errors, which are commonly existed in ANN-to-SNN conversion and propose a scatter-and-gather conversion mechanism for SNNs. This work is based on our previous algorithm (Zou et al., 2020) and hardware (Kuang et al., 2021), and we extend it by (a) testing its robustness on input noise and larger dataset (CIFAR-100), (b) developing a incremental mapping framework to carry out an efficient network deployment on a typical crossbar-based neuromorphic chip, (c) detailed power and speed analyses are given to show its excellent application potential. All together, the main contributions of this article are summarized as follows:

1. Compared with existing ANN-to-SNN conversion methods, the proposed conversion algorithm with quantization constraint can be jointly optimized at training stage, which greatly eliminate the common spike approximation errors. The final accuracy can benefit from higher quantization level and upper bound. Our presented spiking LeNet and VGG-Net achieve great classification accuracies and source code can be available online^1^;
2. An incremental mapping algorithm is presented to optimize network topology placement on a reconfigurable neuromorphic chip with maximum resource efficiency and sufficient flexibility. Besides, three novel evaluation criteria are proposed to analyze resource utilization on general crossbar-based neuromorphic hardware;
3. Experimental results show that our four-spike LeNet and VGG-Net can achieve about 99.37% and 91.91% test accuracy on MNIST and CIFAR-10 dataset, respectively, while our system can obtain nearly 0.38 and 3.24 ms/image real-time inference speed, and an average power consumption of 0.28 and 2.3 mJ/image accordingly. It should be noted that the presented spiking models can be also mapped onto many large-scale neuromorphic platforms like TrueNorth (Akopyan et al., 2015) and BiCoSS (Yang et al., 2021b) built with integrate-and-fire (IF) neurons.

The rest of this article is organized as follows. section 2 introduces the principle of proposed median quantization and scatter-and-gather conversion. In section 3, we introduce a reconfigurable neuromorphic chip and present an incremental mapping workflow to complete model deployment. Experimental results including classification accuracy, resource utilization, and inference speed are presented in section 4. Finally, section 5 concludes this paper.

## 2. Network Conversion

### 2.1. Background

Conventional CNNs are mainly composed of an alternate cascade of convolutional layer, ReLU (Glorot et al., 2011) activation function, and pooling layer. For improving final accuracy and learning efficiency in deep networks, there is usually an additional batch normalization layer located between the convolution layer and ReLU activation function, which achieves an output distribution of zero-mean and unit variance. Used as a standard module in most state of art CNNs, a general convolutional layer can be formulated as Equations (1)–(3):


(1) $$\begin{array}{l} Conv\;s=\underset{i,j,k}{\sum }w_{i,j,k}*x_{i,j,k} \end{array}$$

(2) $$\begin{array}{l} BN\;r=\frac{s-\mu }{\sigma +\varepsilon }+\beta \end{array}$$

(3) $$\begin{array}{l} ReLU\;y=\max\left(0,r\right) \end{array}$$

where *i, j, k* indicate the width, height, and channel dimension of a convolutional kernel, *s* is inner product result of weight *w* and input *x*, μ and σ are the mean and standard deviation of *s*, β is the bias term, ε = 10^−6^ for numerical stability. Note, we omit the scaling term in all equations involved with BN for the convenience of description. Because parameter-free pooling layer is used for simple down-sampling, most of the memory and power budgets come from intensive high-precision (32/64 bits) float-point or fixed-point MAC operations in convolutional layers.

For a spiking neural network built with IF neurons (Abbott, 1999), the membrane potential *V* of each neuron will change due to the spike integration *x* from other neurons *i* at every time step *t* as Equation 4, where *w* represents the synaptic strength. A neuron will emit a spike at some time when its membrane potential is greater than a pre-defined threshold in Equation 5. This discrete spiking dynamic behavior is quite different from ANNs, in which the activation function is continuous.


(4) $$\begin{array}{l} V\left(t+1\right)=V\left(t\right)+\underset{i}{\sum }x_{i}\left(t\right)*w_{i} \end{array}$$

(5) $$\begin{array}{l} Spike=\{\begin{array}{c} 0\;\text{if }V\left(t+1\right)<\theta \\ 1\;\text{if }V\left(t+1\right)\geq \theta \end{array} \end{array}$$

To take advantage of end-to-end training process in deep learning, we are looking forward to an effective method which can convert a quantized and high-accuracy CNN to a spike-based SNN with nearly lossless accuracy. By comparison through the forward process between contemporary CNNs and SNNs, we summarize several key differences as follows:

1. SNN has no individual normalization layer and pooling layer but particular threshold terms θ.
2. SNN usually communicates with timed spike trains of binary value {0,1}, instead of continuous values.
3. If we try an ANN-to-SNN conversion method, how to ensure that firing rate of each spiking neuron is absolutely proportional to corresponding activation output of an ANN neuron without approximation errors.

In this work, we use convolutions with stride of 2 to replace pooling for structure unity, which was proved to be feasible (Springenberg et al., 2014; Esser et al., 2016). Therefore, the main problem is how to deal with incompatible batch normalization layer and continuous activation function, which are essential for a deep ANN training and final accuracy performance.

### 2.2. Training With Median Quantization

**Figure 1:**
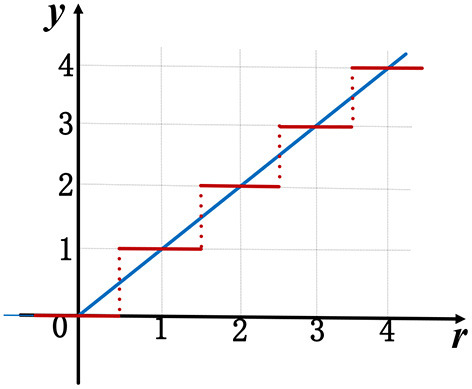
A median quantization with *k* = 0 and *B* = 4 for ReLU. The blue line shows original ReLU function and the red line for the quantized ReLU.

Previous works such as Lee et al. (2016) and Bodo et al. (2017) intend to maintain a balance between the synaptic weights and firing thresholds using a robust normalization method based on maximum value of weights or activations in each layer. However, there are always big spiking approximation errors accumulated in higher layer, which explains why it takes a longer time (dozens or hundreds of time steps) to achieve high correlations of ANN activations. Moreover, the final accuracy and real-time performance of spiking models will seriously suffer from this effect. In contrast, we choose to take these common approximation errors into consideration at model training stage with a median quantization constraint formulated as in Equation (6):


(6) $$\begin{array}{l} Quant\left(r\right)=clip\left(\frac{round\left(r*2^{k}\right)}{2^{k}},0,B\right) \end{array}$$

where *r* is the batch normalization output (Equation 2), and the quantization level *k* and upper bound *B* are two hyper-parameters, which determine the spike encoding precision. For example, when the quantization level *k* = 0 and upper bound *B* = 4, this quantized ReLU (Figure 1) can be formulated as follows:


(7) $$\begin{array}{l} y=\{\begin{array}{l} 2\;if\;r\geq 1.75\\ 1.5\;if\;1.25\leq r<1.75\\ 1\;if\;0.75\leq r<1.25\\ 0.5\;if\;0.25\leq r<0.75\\ 0\;if\;r<0.25 \end{array} \end{array}$$

where *y* is the output of quantized ReLU. Then, we can further integrate batch normalization (Equation 2) into quantization (Equation 7) and modify it as:


(8) $$\begin{array}{l} y^{,}\\ =\{\begin{array}{l} 4\;if\;s^{,}\geq \left(1.75-\beta \right)\left(\sigma^{,}+\varepsilon \right)+\mu^{,}\\ 3\;if\;\left(1.25-\beta \right)\left(\sigma^{,}+\varepsilon \right)+\mu^{,}\leq s^{,}<\left(1.75-\beta \right)\left(\sigma^{,}+\varepsilon \right)+\mu^{,}\\ 2\;if\;\left(0.75-\beta \right)\left(\sigma^{,}+\varepsilon \right)+\mu^{,}\leq s^{,}<\left(1.25-\beta \right)\left(\sigma^{,}+\varepsilon \right)+\mu^{,}\\ 1\;if\;\left(0.25-\beta \right)\left(\sigma^{,}+\varepsilon \right)+\mu^{,}\leq s^{,}<\left(0.75-\beta \right)\left(\sigma^{,}+\varepsilon \right)+\mu^{,}\\ 0\;if\;s^{,}<\left(0.25-\beta \right)\left(\sigma^{,}+\varepsilon \right)+\mu^{,} \end{array} \end{array}$$

(9) $$\begin{array}{l} \mu^{,}=2*\mu ,\;\sigma^{,}=2*\sigma \end{array}$$

where *s*^,^ is the new inner product, together with mean μ^,^ and standard deviation σ^,^ need be scaled twice of original values in Equation (2). Intuitively, the amplitude of quantized ReLU exactly matches spike counts of SNNs. In this example, there are at most four spikes generated. It should be noted that both of the quantization level and upper bound are adjustable as a trade-off between final accuracy and firing rate. Higher quantization level or upper bound may result in a better classification performance but will bring more spikes, which will be discussed in section 4. To enable gradient-based training, we use a straight-through estimator (STE) previously introduced in Bengio et al. (2013), which replaces the piecewise ReLU (red line in Figure 1) with its continuous version (blue line in Figure 1) in backward pass process. Therefore, the above conversion coefficients and accuracy performances can be iteratively optimized with our proposed quantization constraints during training. More specially, the batch normalization operation (Equation 2), which is incompatible with SNNs, can be merged into ReLU activation function without any computing cost^2^.

### 2.3. Conversion With Scatter-and-Gather

**Figure 2:**
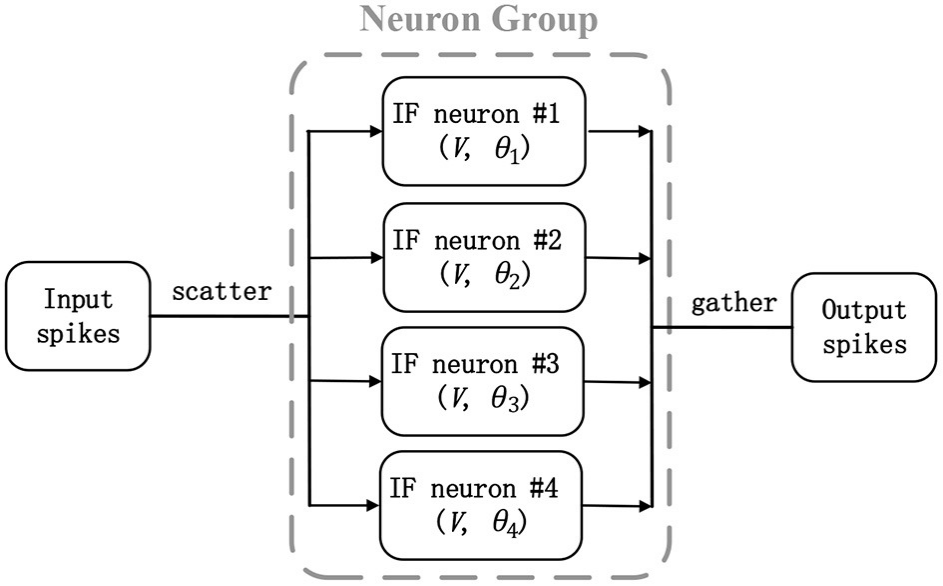
A scatter-and-gather mechanism in artificial neural network (ANN)-to-spiking neural network (SNN) conversion: Four integrate-and-fire (IF) neurons (neuron group) work synchronously and replace an equivalent ANN neuron.

Based on quantized ANNs presented above, we develop a rate-based conversion method called scatter-and-gather for SNNs. For instance of network with quantization level *k* = 1 and upper bound *B* = 2, we need configure four SNN neurons with different thresholds described as in Equation (10) to match the activation output of one ANN neuron, and each spiking neuron will fire at most once within only one time step,


(10) $$\begin{array}{l} \{\begin{array}{l} V\left(t+1\right)=V\left(t\right)+\underset{i}{\sum }x_{i}\left(t\right)*w_{i}\\ \theta_{1}=\mu^{,}+\left(0.25-\beta \right)*\left(\sigma^{,}+\varepsilon \right)\\ \theta_{2}=\mu^{,}+\left(0.75-\beta \right)*\left(\sigma^{,}+\varepsilon \right)\\ \theta_{3}=\mu^{,}+\left(1.25-\beta \right)*\left(\sigma^{,}+\varepsilon \right)\\ \theta_{4}=\mu^{,}+\left(1.75-\beta \right)*\left(\sigma^{,}+\varepsilon \right) \end{array} \end{array}$$

where *V* is the shared membrane potential for four IF neurons, *x* is the incoming spike, *w* is the strength of corresponding synapse which is same as original ANN counterpart, θ is the threshold, and other variables are the batch normalization terms in Equation (8). This converted neuron model is really similar to the McCulloch and Pitts model (Hayman, 1999), where simple threshold gates are enabled and there is no temporal information integration. The only difference is that threshold choices of each neuron may be different. This scatter-and-gather mechanism is described in Figure 2. Four SNN neurons work synchronously, receive the same spike inputs, and share the same synaptic strengths, but fire with respective threshold (θ_1_-θ_4_). Hence, the total time step for one sample simulation will be always 1 and membrane potential will be reset after firing and prepare for next new sample. It should be noted that the proposed scatter-and-gather conversion is really different from AMOS algorithm (Stckl and Maass, 2019). AMOS needs to use different transmitting delays between intra- and inter-layer neurons to maintain information synchronization within multiple time steps. Besides, their conversion coefficients and thresholds are determined by fitting activation gates after ANN training, but our parameter determination method described in Equations (8)–(10) guarantees a lossless conversion from the corresponding quantized ANNs. Compared with Esser et al. (2016), our method can be seen as a generalization from a single spike to multi-spike conversion, to some extent.

## 3. Network Mapping

In this section, we briefly describe the structure and function of a reconfigurable neuromorphic chip (Kuang et al., 2021), and then present an incremental mapping workflow to demonstrate efficient hardware deployments for our converted SNNs.

### 3.1. Neuromorphic Processor

**Figure 3:**
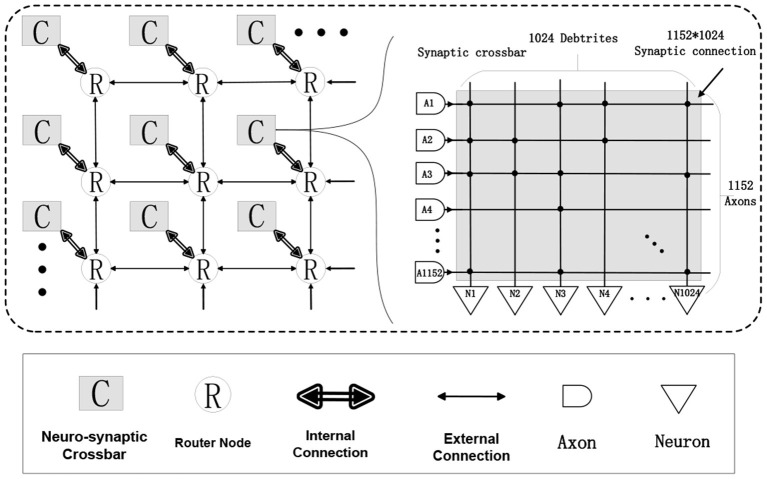
A structural view of our neuromorphic chip: 8 * 8 chips form a multi-chip array, each chip consists of 1,024 LIF neurons, 1,152 axons and a connected synaptic crossbar of 1,152 * 1,024 size.

This chip (Kuang et al., 2021) is designed as a neuro-synaptic processing core, which consists of 1,152 transmission axons, 1,024 basic LIF spiking neurons, and an 1,152 * 1,024 synaptic crossbar (see Figure 3). There is a multicasting router working with an address event representation (AER) protocol (Boahen, 2000) in each chip. The AER router is responsible for receiving and sending signals, which includes general spike packets and programming and test packets. With four AER interfaces in the east, west, north, and south directions, multiple chips can be formed as an 8 * 8 mesh network to support a larger-scale system.

**Figure 4:**
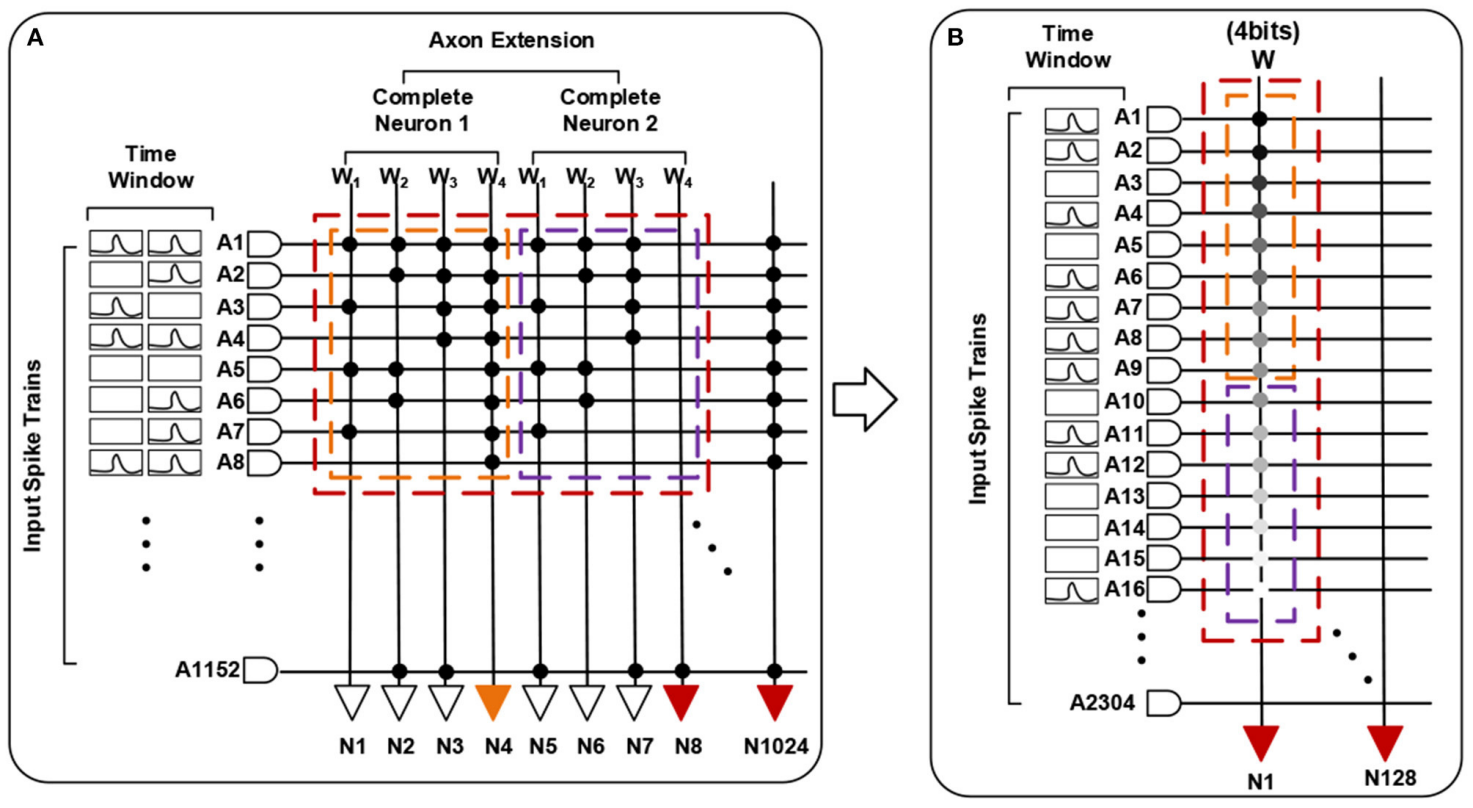
A functional view of our neuromorphic chip. **(A)** describes a spatial neuron with axon extension *f* = 2 (two complete neurons) and a combination for 4-bit weights. **(B)** is the equivalent one.

Each basic spiking neuron has an individual programmable connectivity strength shared by connected 1,152 synapses, each of which can be additionally configured as on or off state. We can employ multiple basic neurons with different connectivity strengths, to make up a complete neuron and achieve a multi-bit (1, 2, 4, 8) weight representation. For example, for a combination of four basic neurons with respective connectivity strength {*w*_1_ = 1, *w*_2_ = 2, *w*_3_ = 4, *w*_4_ = −8}, we can achieve a 4-bit representation range of −8 to 7. Moreover, this neuro-synaptic crossbar supports a spatial axon extension at most 64 (1, 2, 4, 8, 16, 32, 64) times during a complete computing period, to take in a larger feature map input (fan-in) with the cost of decreasing the number of output neuron ports (fan-out) on chip. As illustrated in Figure 4, a spatial neuron is comprised of two complete neurons to support a double (1,152 * 2) fan-in of feature receptive field and the output neuron ports halve. For an extreme instance, we can support a largest convolutional kernel of 3 * 3 * 2,048 and output only one feature point. All in all, these two reconfigurability functions improve the precision of synapses and enhance the ability for processing larger receptive field of convolution and pooling.

In the working mode, the dynamic LIF neuron dynamics behavior is performed and membrane potential is updated. It should be noticed that synaptic nodes which are not triggered by spike events will have no computation activity. Spike events in typical neuromorphic systems are generally discrete and sparse, which can be efficiently delivered by the AER router and multicast among multiple cores. For some larger-scale neural networks exceeding on-chip memory, two alternate SRAMs will work alternately like a ping-pong buffer to enhance the computing throughput. In other words, when memory controller is reading the current weight parameters and neuron states from one of them, a direct-memory-access (DMA) controller will take new programming data (weight parameter and scheduling information) from off-chip memory and writes them to the other one to update the synapse connectivity and neuron states. With the ability of ping-pong reuse, our chip should have potential to implement large-scale network architectures like VGG-Net (Simonyan and Zisserman, 2014) on one core, compared with many other large-scale neuromorphic chips (Akopyan et al., 2015; Yang et al., 2021b) in general.

### 3.2. Mapping Strategy

**Figure 5:**
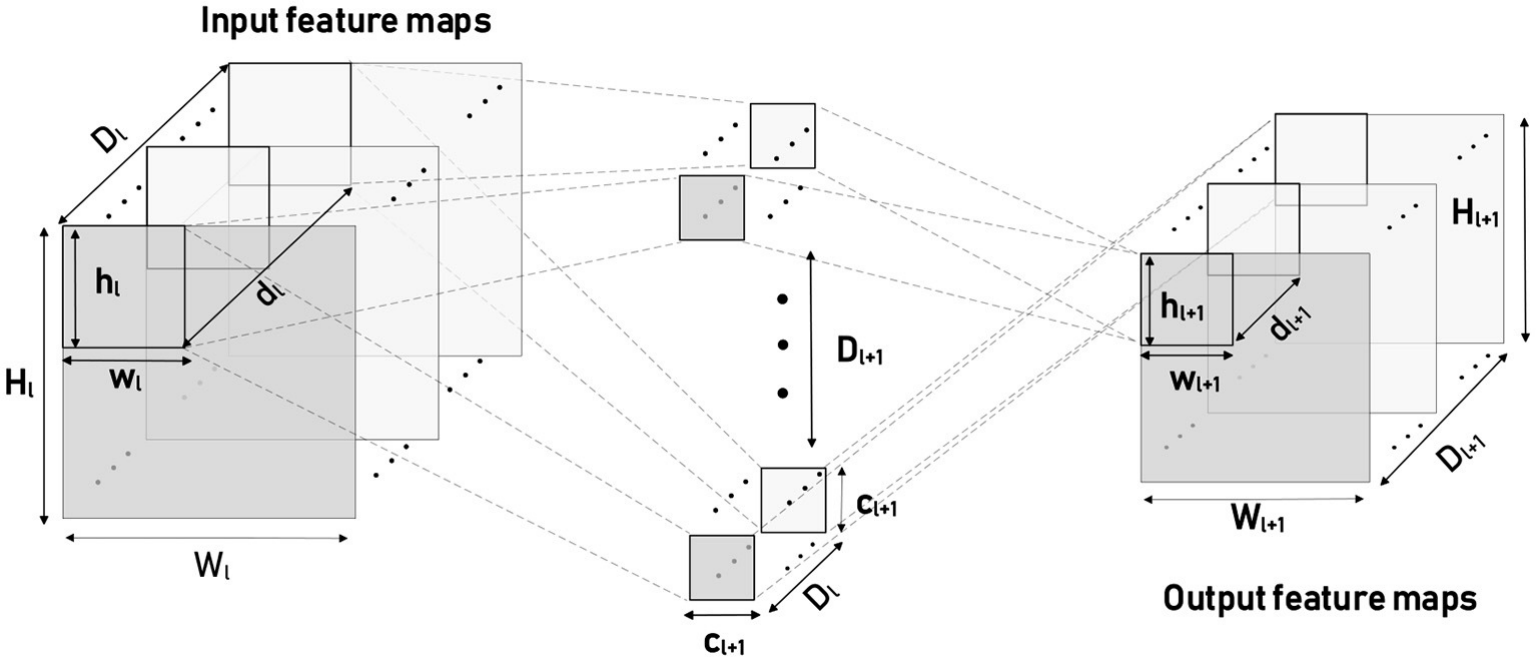
Input and output patches on the corresponding feature maps.

However, almost all contemporary neuromorphic hardwares, designed with 2D crossbar-based structure, have typical block-wise constraints for neuron connectivity (Bouvier et al., 2019). For a standard 2-D crossbar unit with finite inputs and outputs (256 * 256 for TrueNorth), it is impossible to process a complete convolutional layer individually. Building with 256 * 256 synaptic computing core, TrueNorth has to use group convolution (Esser et al., 2016) to cut a large convolutional layer into many slices. For the sake of description, we adopt a series of definitions in Table 1 for different notations. Due to local speciality of convolution operation, a common approach is to partition 3-D input feature maps into a number of *m* * *n* patches seeing (Figure 5) to ensure that the size of each patch is less than the number of input axons. In this case, each patch is a spatial topographic location involving all of input feature map channels in Equation (12). Adjacent patches have a specific overlapping region that depends on the kernel size and stride of convolution or pooling. In contrast, our chip could extend processing receptive field for a larger input patch with larger width or height by reusing 1,152 input axons for *f* times in Equation (11).


(11) $$\begin{array}{l} w_{l}*h_{l}*d_{l}\leq \frac{1,152*f}{k_{rep}},\;w_{l+1}*h_{l+1}*d_{l+1}\leq \frac{1,024}{f*k_{wei}*k_{rep}} \end{array}$$

(12) $$\begin{array}{l} d_{l}=D_{l},\;d_{l+1}\leq D_{l+1} \end{array}$$

(13) $$\begin{array}{l} w_{l+1}=\frac{w_{l}-c_{l+1}}{s_{l+1}}+1,\;h_{l+1}=\frac{h_{l}-c_{l+1}}{s_{l+1}}+1 \end{array}$$

Accordingly, the size of resulted output patch can be calculated from the size of input patch as in Equation (13). As introduced in previous section, the output size may be greater than the number of fewer output neurons, because of higher weight precisions or spatial axon extension. For example, if we want to implement a 1-bit convolution (*k*_*rep*_ = 1, *k*_*wei*_ = 2) for an input feature maps of 4 * 4 * 128 (*W*_*l*_ * *H*_*l*_ * *D*_*l*_) with a filter kernel of 2 * 2 * 128 * 256 (*c*_*l*+1_ * *c*_*l*+1_ * *D*_*l*_ * *D*_*l*+1_) and stride of 2 (*s*_*l*+1_ = 2), the size of output feature maps can be calculated as 2 * 2 * 256 (*D*_*l*+1_ * *H*_*l*+1_ * *D*_*l*+1_) according to Equation (13). Then, there may be two mapping options: 1: four identical input patches of 4 * 4 * 128 (*w*_*l*_ * *h*_*l*_ * *d*_*l*_) distributed on four computing cores, respectively; each of which contributes to an output patch of 2 * 2 * 64 (*w*_*l*+1_ * *w*_*l*+1_ * *w*_*l*+1_); 2: two complementary input patches of 4 * 2 * 128 distributed on two computing cores, respectively; each of which contributes to an output patch of 2 * 1 * 256. Detailed mapping results are shown in Table 2.

**Table 1:** Summary of main notations.

**Notation**	**Description**
W	Width of input/output feature map
H	Height of input/output feature map
D	Depth of input/output feature map
w	Width of input/output patch
h	Height of input/output patch
d	Depth of input/output patch
l	*l*th layer of convolution/pooling
m	Number of horizontal patches
n	Number of vertical patches
p	Number of depth-oriented patches
c	Kernel size of convolution/pooling
s	Stride of convolution/pooling
*k*_*rep*_ ^a^	How many SNN neurons replace an ANN neuron
*k*_*wei*_ ^b^	Bit-width of weight parameter
f	Axon extension
core	Neuro-synaptic crossbar
patch	Partial feature map
time step	Computing time for all neurons on a core

a For spatial conversion, an ANN neuron will be replaced by multiple SNN neurons, i.e., k_rep_ = B^*^2^k^.

b *k_wei_ represents the quantization bit-width for weight parameters of convolution kernels. In this article, we fixed k_wei_ = 2 for a simple ternary quantization of {–1, 0, +1}*.

**Table 2:** Mapping results of two plans.

**Plan**	***w*_*l*_**	***h*_*l*_**	***d*_*l*_**	***w*_*l*+1_**	***h*_*l*+1_**	***d*_*l*+1_**	***f***	***core***
No. 1	4	4	128	2	2	64	2	4
No. 2	4	2	128	2	1	256	1	2

Here, we define three practical evaluation criteria (Equations 14–16) to thoroughly figure out how many effective axons, neurons, and synapse connections are occupied in a standard neuro-synaptic crossbar. Higher utilization density means a more compact mapping with less resource consumption and reduces redundancy.


(14) $$\begin{array}{l} Density_{neuron}=\frac{k_{rep}*\left(w_{l+1}*h_{l+1}*d_{l+1}\right)*f*k_{wei}}{1,024} \end{array}$$

(15) $$\begin{array}{l} Density_{axon}=\frac{k_{rep}*\left(w_{l}*h_{l}*d_{l}\right)}{1,152*f} \end{array}$$

(16) $$\begin{array}{l} Density_{synapse}\\ =\frac{k_{rep}*\left(w_{l+1}*h_{l+1}*d_{l+1}\right)*\left(c_{l+1}^{2}*d_{l}*k_{rep}*k_{wei}\right)}{1,152*1,024} \end{array}$$

We summarize the three criteria of two plans in Table 3. It can be found that both of the *Density*_*neuron*_ and *Density*_*axon*_ are the same, but *Density*_*synapse*_ of the No. 2 is twice as high as that of the No.1. From a hardware perspective, worse utilization of crossbar will lead to a more resource budget and multicast communication workload for fixed sized feature maps. Hence, there is a tradeoff between the size of input patch and output patch. Larger width or height of patches does not mean better resource efficiency on a specific chip. For each patch on a neuro-synaptic crossbar, *d*_*l*_,*c*_*l*+1_,*k*_*rep*_,*k*_*wei*_ are all constant, we need to selectively increase *w*_*l*_,*h*_*l*_,*f* or *d*_*l*+1_ for a maximum utilization of axon, neuron, and synapse. Learning from the example above, a progressive strategy is to give the priority to increase output channel *d*_*l*+1_ and do not increase *w*_*l*_ and *h*_*l*_ until *d*_*l*+1_ is up to *D*_*l*+1_, while there are still available neurons for axon extension. This priority leads to the minimum overlapping chance of sliding windows along width and height and guarantees all of hardware modules are working with a high resource efficiency.

**Table 3:** Resource efficiency of two plans.

**Plan**	***Density*_*neuron*_**	***Density*_*axon*_**	***Density*_*synapse*_**
No. 1	100%	89%	22%
No. 2	100%	89%	44%

**Figure 6:**
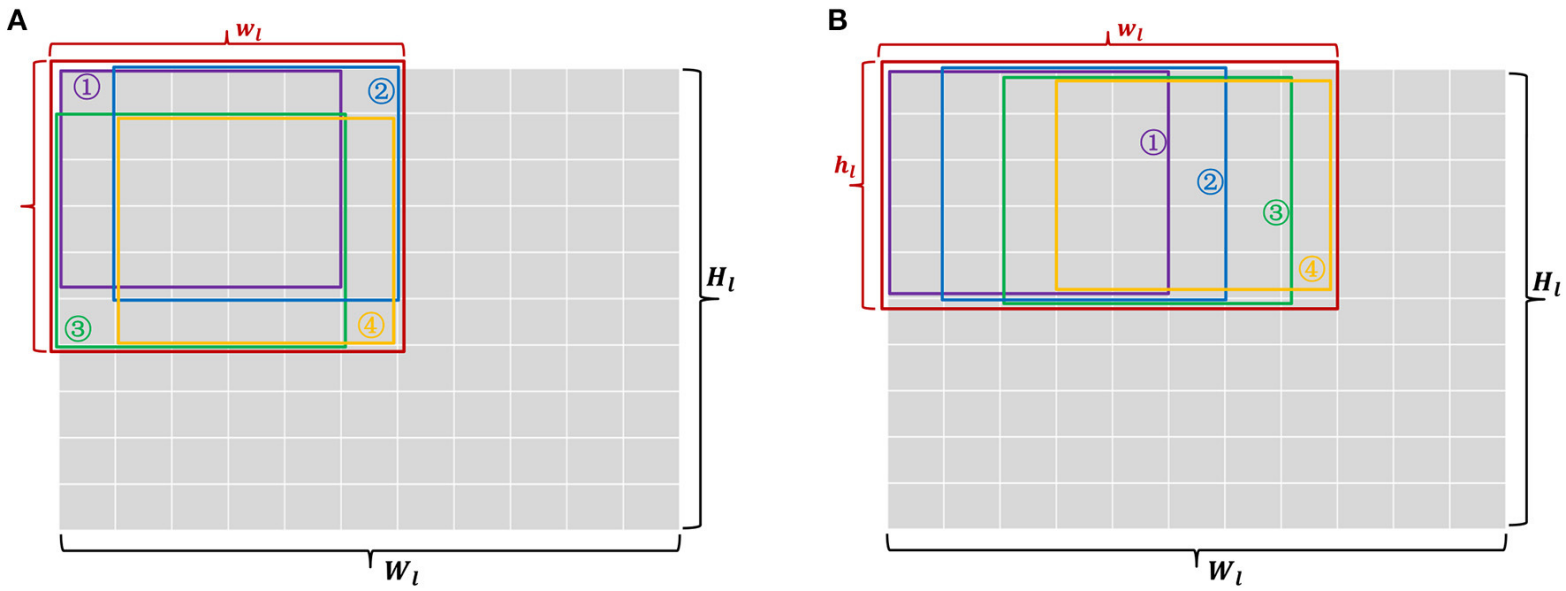
Two kinds of input patches **(A,B)** which generate the same sized output patches. But the shape is square in **(A)** and rectangular in **(B)**. Each colored box denotes a 5 * 5 receptive field of convolution, except the red box that denotes a total input patch.

After the primary size of each patch is determined, another problem is how to choose the shape. We can take an intuitive understanding in Figure 6. It shows output patches with the same size (2 * 2 and 1 * 4) may have multiple options to be generated from different sized input patches. Similarly, input patches with the same size but different shapes will generate different number of sliding windows, which means the size of output patches are not equal. We can consider the mean-value inequality for *w*_*l*_,*h*_*l*_ seeing (Equation 17). The equality become valid only when *w*_*l*_ = *h*_*l*_. The size of input and output patch is proportional to the product result on the left of Equations (17) and (18). If *w*_*l*_ * *h*_*l*_ is a constant, maximizing *w*_*l*+1_ * *w*_*l*+1_ must require *w*_*l*_ = *h*_*l*_. This means a square patch is more compact than rectangular one and should be the first choice.


(17) $$\begin{array}{l} w_{l}*h_{l}\leq \frac{{\left(w_{l}+h_{l}\right)}^{2}}{4} \end{array}$$

(18) $$\begin{array}{l} w_{l+1}*h_{l+1}=\left(\frac{w_{l}-c_{l+1}}{s_{l+1}}+1\right)*\left(\frac{h_{l}-c_{l+1}}{s_{l+1}}+1\right)\\ =\frac{w_{l}+h+\left(w_{l}+h_{l}\right)*\left(s_{l+1}-c_{l+1}\right)+{\left(s_{l+1}-c_{l+1}\right)}^{2}}{s_{l+1}^{2}} \end{array}$$

**Figure 7:**
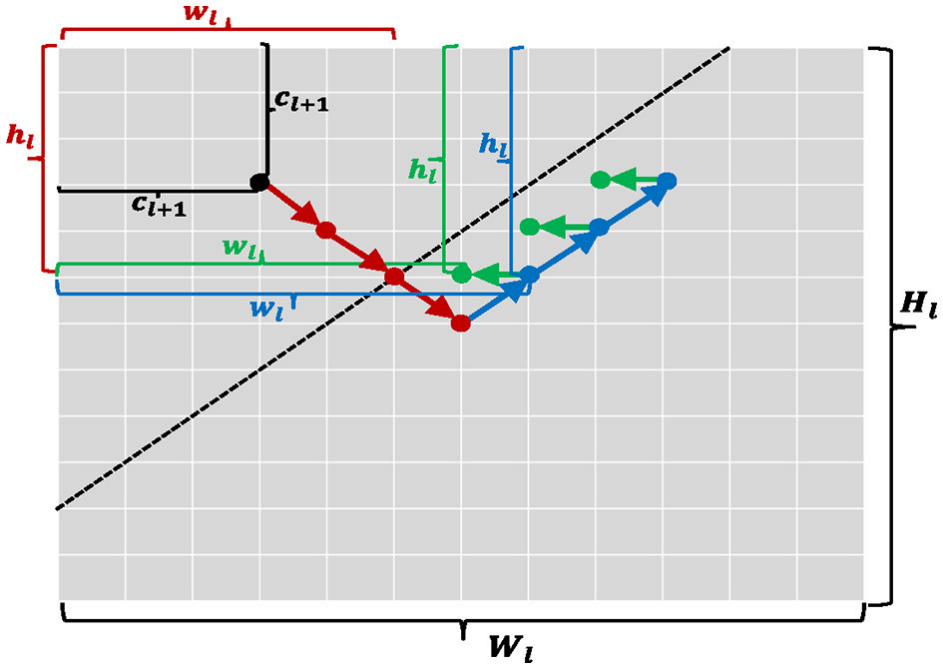
A graphical description for Algorithms 1 and 2. A step-by-step grid search is performed and arrows with different colors denote different search directions. Each of colored dots is a candidate item of parameter configurations.

For an overall consideration of patch size and shape, a channel-major and square-major mapping algorithm is described in Algorithms 1, 2 and Figure 7, respectively. We integrate above two priority principles into a progressive grid search strategy to obtain an optimal choices for undetermined parameters, i.e., a list of *w*_*l*_, *w*_*l*_, *w*_*l*_, *w*_*l*+1_, *h*_*l*+1_, *d*_*l*+1_, and *f*. In Algorithm 1, we first initialize each parameter with minimum, and then Algorithm 1 would gradually increase the number of patch channels (*d*_*l*+1_) but fix the patch width and height (*w*_*l*+1_, *h*_*l*+1_) until *d*_*l*+1_ equals *D*_*l*+1_ or the output neurons on a core are used up. Last but not least, if there are still remaining resources unused after Algorithm 1 procedure, Algorithm 2 will perform a step-by-step multi-path grid search process for potential and feasible mapping choices and output the maximum one for target crossbar-based neuromorphic chip.

**Algorithm 1:** Channel-major search.

This is the first procedure to generate an primary input and output patch size including (*w*_*l*_, *h*_*l*_, *d*_*l*_, *w*_*l*+1_, *h*_*l*+1_, *d*_*l*+1_, *f*). The output channel *d*_*l*+1_ will be less than or equal to *D*_*l*+1_.
**Require:** quantization precisions (*k*_*rep*_, *k*_*wei*_), kernel size (*c*_*l*+1_) and the number of output feature map channels (*D*_*l*+1_)
**Ensure:** primary input and output patch size including (*w*_*l*_, *h*_*l*_, *d*_*l*_, *w*_*l*+1_, *h*_*l*+1_, *d*_*l*+1_, *f*)
1: **Initialize:** *w*_*l*_ = *c*_*l*+1_, *h*_*l*_ = *c*_*l*+1_, *d*_*l*_ = *D*_*l*_, *f* = 1;
2: **for** *d*_*l*+1_ = 1 to *D*_*l*+1_ **do**
3: **if** meet the left of constraint (Equation 11) **then**
4: ;
5: **else**
6: *f* = *f* * 2, jump to line 3;
7: **end if**
8: **if** not meet the right of constraint (Equation 11) **then**
9: output (*w*_*l*_, *h*_*l*_, *d*_*l*_, *w*_*l*+1_, *h*_*l*+1_, *d*_*l*+1_−1, *f*);
10: **else**
11: ;
12: **end if**
13: **if** *d*_*l*+1_ = *D*_*l*+1_ **then**
14: output (*w*_*l*_, *h*_*l*_, *d*_*l*_, *w*_*l*+1_, *h*_*l*+1_, *d*_*l*+1_, *f*);
15: **end if**
16: **end for**

**Algorithm 2:** Square-major search.

This is the second procedure that should be executed after Algorithm 1 and when *d*_*l*+1_ = *D*_*l*+1_, and obtain a final input and output patch size including (*w*_*l*_, *h*_*l*_, *d*_*l*_, *w*_*l*+1_, *h*_*l*+1_, *d*_*l*+1_, *f*).
**Require:** quantization precisions (*k*_*rep*_, *k*_*wei*_), kernel size (*c*_*l*+1_) and the width and height of input feature map (*W*_*l*_, *H*_*l*_)
**Ensure:** final input and output patch size including (*w*_*l*_, *h*_*l*_, *d*_*l*_, *w*_*l*+1_, *h*_*l*+1_, *d*_*l*+1_, *f*)
1: **for** *w*_*l*_ = *c*_*l*+1_ to *W*_*l*_ or *h*_*l*_ = *c*_*l*+1_ to *H*_*l*_ **do**
2: take a red step in Figure 7;
3: **if** meet the left of constraint (Equation 11) **then**
4: **if** meet the right of constraint (Equation 11) **then**
5: ;
6: **else**
7: take a bluestep in Figure 7;
8: **if** meet the right of constraint (Equation 11) **then**
9: mark (*w*_*l*_, *h*_*l*_, *d*_*l*_, *w*_*l*+1_, *h*_*l*+1_, *d*_*l*+1_, *f*)
10: **else**
11: take a green step in Figure 7;
12: **if** meet the right of constraint (Equation 11) **then**
13: mark (*w*_*l*_, *h*_*l*_, *d*_*l*_, *w*_*l*+1_, *h*_*l*+1_, *d*_*l*+1_, *f*)
14: **else**
15: take a blue step in Figure 7;
16: **if** meet the right of constraint (Equation 11) **then**
17: mark (*w*_*l*_, *h*_*l*_, *d*_*l*_, *w*_*l*+1_, *h*_*l*+1_, *d*_*l*+1_, *f*)
18: **else**
19: jump to line 11;
20: **end if**
21: **end if**
22: **end if**
23: **end if**
24: compare all marks and output the maximum, exit;
25: **else**
26: **if** *f* == 64 **then**
27: output (*w*_*l*_, *h*_*l*_, *d*_*l*_, *w*_*l*+1_, *h*_*l*+1_, *d*_*l*+1_, *f*);
28: **else**
29: *f* = *f* * 2, jump to line 3;
30: **end if**
31: **end if**
32: **end for**

### 3.3. Spatial Mapping

**Figure 8:**
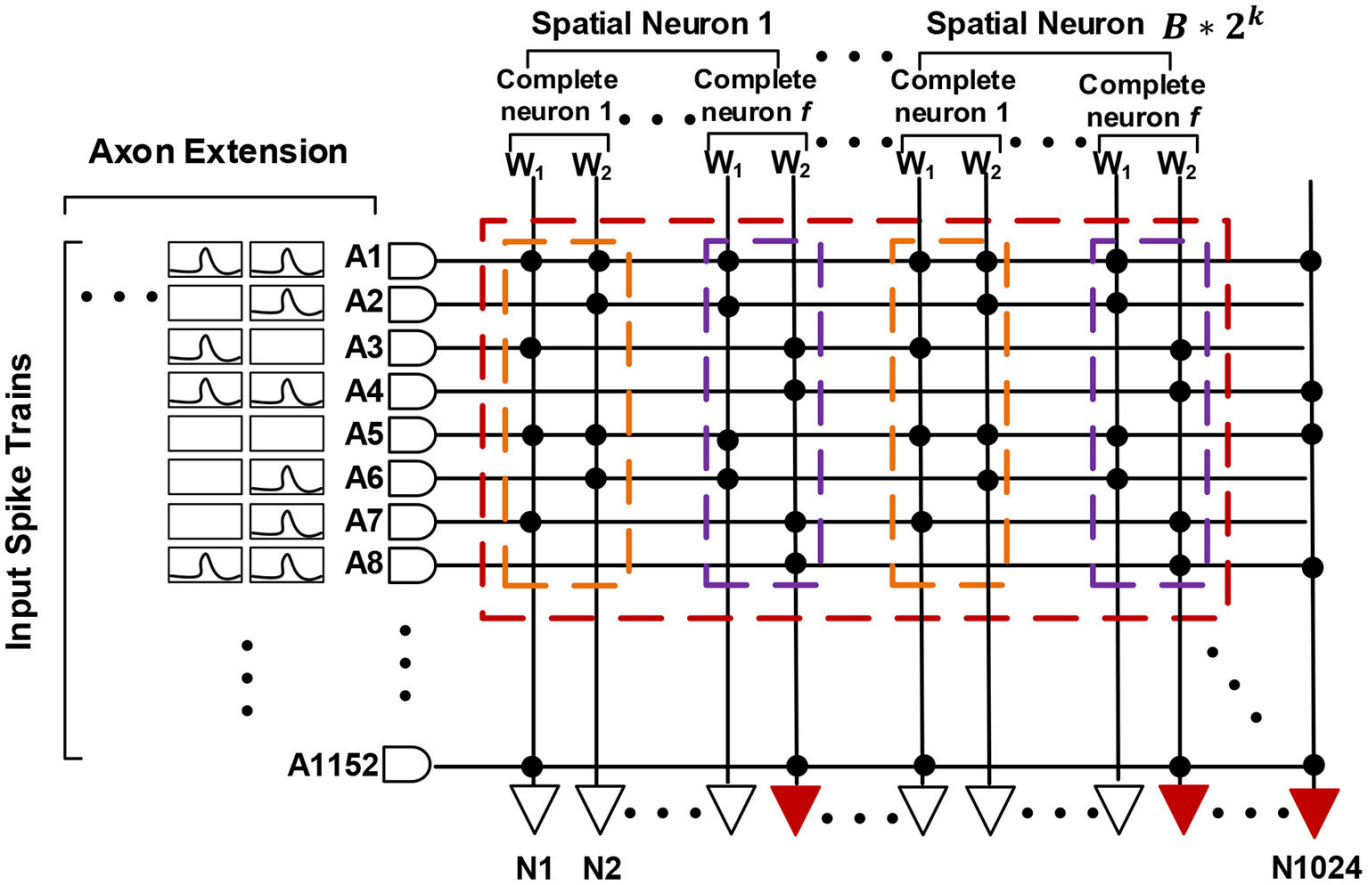
Spatial mapping for spiking neural networks (SNNs) with scatter-and-gather conversion. An artificial neural network (ANN) neuron is replaced by *B* * 2^*k*^ spatial SNN neurons, and each spatial neuron comprises *f* complete neurons with ternary-valued weights.

As mentioned above, in this work, we mainly use a simple ternary-valued {−1,0,+1} weight quantization. Therefore, for an SNN with different spike encoding precisions (*k* and *B*), we can configure *k*_*rep*_ and *k*_*wei*_ as follows:


(19) $$\begin{array}{l} k_{rep}=B*2^{k},\;k_{wei}=2 \end{array}$$

where *k*_*rep*_ means an ANN neuron is replaced by *B* * 2^*k*^ SNN spatial neurons, each of which has the same synaptic connections and spike inputs but fire with different thresholds as discussed in section 2; *k*_*wei*_ means each complete neuron is composed of two basic spiking neurons with respective weight {*w*_1_ = -1, *w*_2_ = 1} as in Figure 8. The number (*f*) of complete neurons contained in a spatial neuron is determined by the size of feature maps and *k*_*rep*_ according to Algorithms 1, 2. For a complete convolutional or pooling layer, if we keep each patch equal, the numbers of horizontal, vertical and depth-oriented patches would be calculated as Equations (20)–(22), respectively.


(20) $$\begin{array}{l} W_{l}=w_{l}*m_{l}-\left(m_{l}-1\right)*\left(c_{l+1}-s_{l+1}\right) \end{array}$$

(21) $$\begin{array}{l} H_{l}=h_{l}*n_{l}-\left(n_{l}-1\right)*\left(c_{l+1}-s_{l+1}\right) \end{array}$$

(22) $$\begin{array}{l} D_{l+1}=p_{l}*d_{l+1} \end{array}$$

Finally, we can distribute a total *m*_*l*_ * *n*_*l*_ * *p*_*l*_ convolution patches onto our multi-chip (8 * 8) system on schedule, together with ping-pong working mode. If resources are sufficient, fully unfolded mapping can achieve highest throughput and power efficiency, because the scatter-and-gather conversion ensures all spike signals are accessible at one computing time step for a layer. More importantly, expensive off-chip memory access budgets can be saved.

## 4. Experiments

In this section, we first conduct an ablation study about quantization level *k* and upper bound *B* to evaluate the effectiveness of our proposed conversion and quantization algorithm on MNIST and CIFAR-10/100 dataset using LeNet and VGG-Net architecture, respectively. Then, we carry out practical mapping of above spiking networks onto our neuromorphic system and provide corresponding speed and power analysis results.

### 4.1. Benchmark Applications

1. *MNIST dataset*
  The MNIST dataset (Lecun and Bottou, 1998) of handwritten digit has been widely applied in image classification field, which was collected from postal codes, including a training set of 60,000 examples, and a test set of 10,000 examples. Each example is an individual 28 * 28 pixel grayscale image labeled 0–9. Pixel values are integer (0–255), where 0 means background (white) and 255 means foreground (black). We adopt a classical LeNet (Lecun and Bottou, 1998) architecture (16*C*5-16*C*5-2*P*2-32*C*5-2*P*2-256*FC*-10*FC*)^3^ for this task.
2. *CIFAR-10/100 dataset*
  The CIFAR-10 dataset (Krizhevsky and Hinton, 2009) consists of 60,000 32 * 32 pixel color images in 10 classes, with 6,000 images per class. There are 50,000 training images and 10,000 test images. The CIFAR-100 dataset^4^ is just like the CIFAR-10 but more challenging. It has 100 classes containing 600 images each. There are 500 training images and 100 testing images per class. A VGG-Net (Simonyan and Zisserman, 2014) variant with 13 layers (64*C*3-64*C*3-64*C*3-2*P*2-128*C*3-128*C*3-2*P*2-256*C*3-256*C*3-2P2 -512*C*3-512*C*3-10*FC*) is designed for these two image classification tasks. No data augmentation is used other than standard random image flipping and cropping for training. Test evaluation is based solely on central 24 * 24 crop from test set (for both CIFAR-10 and CIFAR-100).

In our experiments, we use a ternary-valued {-1,0,1} weight quantization as in Li and Liu (2016), not full precision (16 or 32 bits) like many others (Lee et al., 2016, 2020; Bodo et al., 2017; Mostafa et al., 2017; Rueckauer and Liu, 2018; Wu et al., 2018; Yousefzadeh et al., 2019), to facilitate hardware deployment, because we find the weight quantization with more bit-width contributes very little to final accuracy, which is consistent with (Rastegari et al., 2016; Zhou et al., 2016). All convolutional networks are trained using standard ADAM rule (Kingma and Ba, 2014) with an initial learning rate set to 0.001 and 10 times decayed per 200 epochs, based on TensorLayer (Dong et al., 2017), a customized deep learning library. We did not use any weight or spike penalty or dropout (Srivastava et al., 2014) during training.

### 4.2. Quantization Precision

Here, we conduct a series of ablation experiments on two hyper-parameters, i.e., quantization level *k* and upper bound *B*, both of which jointly determine how many spikes each neuron will fire at most and relate to overall resource, latency, and power consumption on hardware. In fact, choosing a proper quantization level and upper bound for a specific network is completely subjective, because less spikes with a low-precision quantization inevitably result in a bigger accuracy loss.

**Figure 9:**
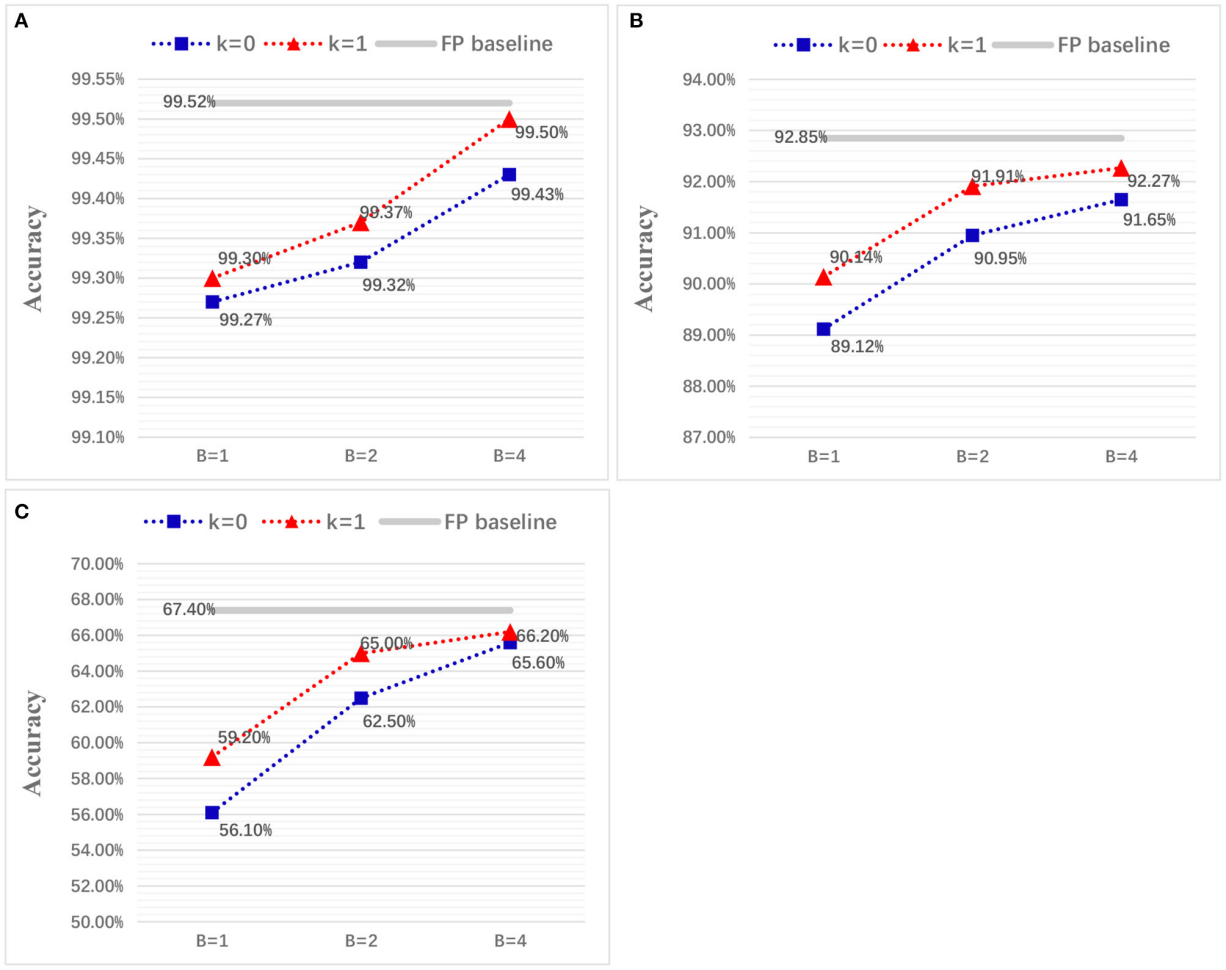
Classification accuracy for LeNet on MNIST **(A)** and VGG-Net on CIFAR-10/100 dataset **(B,C)**, with different quantization precisions.

Considering a successive combination of quantization level *k* in {0,1} and upper bound *B* in {1,2,4}, we report six different test accuracies for LeNet on MNIST and VGG-Net on CIFAR-10/100 (Figure 9). It should be noted we choose not quantize the first and last layer because they are usually used for an image-to-spike encoding and loss calculation as in Esser et al. (2016). Experimental results show that the final accuracy can benefit from both of higher quantization level and upper bound. More importantly, we find that the spiking LeNet and VGG-Net with quantization level *k* = 1 and upper bound *B* = 4 are on a par with their full-precision (FP) baselines. For a comparison with other works, we summarize our results (for *k* = 1, *B* = 2) and many state-of-the-art works in Tables 4–7. It shows that our proposed spiking models are lossless with their quantized ANN counterparts and able to achieve great performance on MNIST among other works, and even better on CIFAR-10/100 dataset. In all experiments except for full-precision baseline, both of weights and activations adopt a low-precision quantization not full-precision (16 or 32 bits) like many others (Bodo et al., 2017; Xu et al., 2017). On the contrary, using this low-precision quantization does not harm to the final accuracy, but enables a cheap memory budget on many popular neuromorphic systems such as Akopyan et al. (2015), Davies et al. (2018), and Kuang et al. (2021). More specially, our networks complete simulation for one input sample within only one time step, compared with other conversion methods with dozens even hundreds of simulation time steps (Lee et al., 2016, 2020; Bodo et al., 2017; Mostafa et al., 2017; Xu et al., 2017; Rueckauer and Liu, 2018; Wu et al., 2018; Yousefzadeh et al., 2019).

**Table 4:** Classification accuracy on MNIST.

	**Activation quantization**	**ANN**	**SNN**
**This work (Full precision)**	**N**	**99.52%**	**N/A**
**This work (Rate coding)**	***k*** **=** **1**, ***B*** **=** **2**	**99.37%**	**99.37%**
**This work (10% noise)**	***k*** **=** **1**, ***B*** **=** **2**	**99.37%**	**99.39%**
**This work (20% noise)**	***k*** **=** **1**, ***B*** **=** **2**	**99.37%**	**99.22%**
Mostafa et al. (2017) (Temporal coding)	N	98.50%	96.98%
Rueckauer and Liu (2018) (Temporal coding)	N	98.96%	98.57%
Wu et al. (2018) (Rate coding)	N	N/A	99.42%
Yousefzadeh et al. (2019) (Rate coding)	N	99.21%	99.19%
Bodo et al. (2017) (Rate coding)	N	99.44%	99.44%

*The bold values are our experimental results*.

**Table 5:** Classification accuracy on CIFAR-10.

	**Activation quantization**	**ANN**	**SNN**
**This work (Full precision)**	**N**	**92.85%**	**N/A**
**This work (Rate coding)**	***k*** **=** **1**, ***B*** **=** **2**	**91.91%**	**91.91%**
**This work (10% noise)**	***k*** **=** **1**, ***B*** **=** **2**	**91.91%**	**90.32%**
**This work (20% noise)**	***k*** **=** **1**, ***B*** **=** **2**	**91.91%**	**89.65%**
Esser et al. (2016) (Rate coding)	1-bit	N/A	89.32%
Bodo et al. (2017) (Rate coding)	N	88.87%	88.82%
Lee et al. (2016) (Rate coding)	N	85.97%	83.54%
Lee et al. (2020) (Rate coding)	N	91.98%	90.54%

*The bold values are our experimental results*.

**Table 6:** Classification accuracy on CIFAR-100.

	**Activation quantization**	**ANN**	**SNN**
**This work (Full precision)**	**N**	**67.4%**	**N/A**
**This work (Rate coding)**	***k*** **=** **1**, ***B*** **=** **2**	**65.0%**	**65.0%**
**This work (10% noise)**	***k*** **=** **1**, ***B*** **=** **2**	**65.0%**	**63.93%**
**This work (20% noise)**	***k*** **=** **1**, ***B*** **=** **2**	**65.0%**	**62.25%**
Esser et al. (2016) (Rate coding)	1-bit	N/A	65.48%

*The bold values are our experimental results*.

**Table 7:** Mapping results for LeNet.

	**Single-spike**						**Two-spike**						**Four-spike**					
	**Input patch**	**Output patch**	**f**	**A^*b*^**	**N**	**S**	**Input patch**	**Output patch**	**f**	**A**	**N**	**S**	**Input patch**	**Output patch**	**f**	**A**	**N**	**S**
16C5^*a*^	9 * 8 * 16	5 * 4 * 16	1	1.0	0.63	0.22	8 * 6 * 16	4 * 2 * 16	2	0.67	1.0	0.35	6 * 6 * 16	2 * 2 * 16	2	1.0	1.0	0.69
2P2	8 * 8 * 16	4 * 4 * 16	1	0.89	0.50	0.03	6 * 6 * 16	3 * 3 * 16	1	1.0	0.56	0.06	4 * 4 * 16	2 * 2 * 16	1	0.89	0.5	0.11
16C5	8 * 8 * 16	4 * 4 * 32	1	0.89	1.0	0.35	6 * 6 * 16	2 * 2 * 32	1	1.0	0.5	0.35	6 * 5 * 16	2 * 1 * 32	2	0.83	1.0	0.69
2P2	4 * 8 * 32	2 * 4 * 32	1	0.89	0.5	0.03	4 * 4 * 32	2 * 2 * 32	1	0.89	0.5	0.06	4 * 2 * 32	2 * 1 * 32	1	0.89	0.5	0.22
256FC	4 * 4 * 32	1 * 1 * 256	1	0.44	0.5	0.22	4 * 4 * 32	1 * 1 * 256	1	0.89	1.0	0.89	4 * 4 * 32	1 * 1 * 64	2	0.89	1.0	0.89
10FC	1 * 1 * 256	1 * 1 * 10	1	0.22	0.02	0.01	1 * 1 * 256	1 * 1 * 10	1	0.44	0.04	0.02	1 * 1 * 256	1 * 1 * 10	1	0.89	0.08	0.07

*The first and last layer are usually processed off chip and not considered here*.

a *Layer is described as output channels-layer type-kernel size, where C is convolution, P is pooling and FC is the fully connected layer*.

b *The initial abbreviation A, N, and S refer to three evaluation criteria including Density_axon_, Density_neuron_ and Density_synapse_ introduced in section 3*.

For evaluation on robustness, we impose two different levels of noises (10%, 20%) on the neurons of input layer. More specifically, the IF neuron branches in Figure 2 will be randomly shut down and never give spike outputs. This robustness evaluation is very similar to the Dropout technique (Srivastava et al., 2014), but we use it at network inference stage. We test LeNet on MNIST and VGG-Net CIFAR-10/100 with quantization precision *k* = 1 and *B* = 2 as in Tables 4–7. It shows our spiking networks are robust enough to tolerate broken neurons (input layer with noises) with maximum degradation of 3% when noise ratio is up to 20%. For noise at 10% level, our spiking LeNet even shows a slightly better accuracies.

### 4.3. Mapping Results

For verifying effectiveness of our mapping algorithm, we carry out practical mapping for spiking LeNet and VGG-Net with various spike encoding precisions onto our neuromorphic chip. The mapping results of spiking LeNet and VGG-Net are summarized in Tables 6, 7. As a convention, we denote the networks with the configurations of {*k* = 0, *B* = 1}, {*k* = 0, *B* = 2}, and {*k* = 1, *B* = 2} as single-spike, two-spike, and four-spike model, respectively. From the two tables, it can be found either different quantization precisions or model sizes show different resource utilization while both spiking LeNet and VGG-Net with higher spike encoding precisions bring linearly better resource efficiency. For spatial mapping of scatter-and-gather SNNs, it is easy to understand that the input and output spike representation with higher precision mean a smaller patch height *h*_*l*_ and width *w*_*l*_ and increase effective synaptic connections for each output neuron, but total patch number, i.e., *m*_*l*_ * *n*_*l*_ * *p*_*l*_ would be bigger.

For LeNet convolutional layer with dozens of channels, the height (*h*_*l*_) and width (*w*_*l*_) of each patch are much bigger than the kernel size, because all of the output channels (*d*_*l*+1_) can be placed on one neuro-synaptic core according to Algorithm 1. Also, it can be seen that the shape of patches are not square occasionally, which can be explained by a balance between *w*_*l*_,*h*_*l*_ and *d*_*l*+1_ discussed in Algorithm 2. For example, the first layer of two-spike LeNet choose an input patch of 8 * 6 * 16 instead of 7 * 7 * 16 or 6 * 6 * 16 as the final mapping plan to attain fine-tuned resource efficiency. This is because the same area (the product of *w*_*l*_, *h*_*l*_) of input patch with unequal height and width (*w*_*l*_≠*h*_*l*_) results in a smaller area (the product of *w*_*l*+1_,*h*_*l*+1_) for output patch but allow a bigger capacity to hold all of output channel (*d*_*l*+1_ = *D*_*l*+1_). This tradeoff is more explicit in VGG-Net with hundreds of channels. Table 8 shows that the channel-major and square-major priorities acquire a more symmetric mapping, where the height and width of each patch are usually equal to kernel size (*c*_*l*+1_). Although each patch cannot contain all output channels (*d*_*l*+1_<*D*_*l*+1_), the mapping algorithm improves the overall resource efficiency (*Density*_*synapse*_) compared with LeNet.

**Table 8:** Mapping results for VGG-Net.

	**Single-spike**						**Two-spike**						**Four-spike**					
	**Input patch**	**Output patch**	**f**	**A**	**N**	**S**	**Input patch**	**Output patch**	**f**	**A**	**N**	**S**	**Input patch**	**Output patch**	**f**	**A**	**N**	**S**
64C3	4 * 4 * 64	2 * 2 * 64	1	0.89	0.5	0.25	4 * 3 * 64	2 * 1 * 64	2	0.67	1.0	0.5	3 * 3 * 64	1 * 1 * 64	2	1.0	1.0	1.0
64C3	4 * 4 * 64	2 * 2 * 64	1	0.89	0.5	0.25	4 * 3 * 64	2 * 1 * 64	2	0.67	1.0	0.5	3 * 3 * 64	1 * 1 * 64	2	1.0	1.0	1.0
2P2	4 * 4 * 64	2 * 2 * 64	1	0.89	0.5	0.11	4 * 2 * 64	2 * 1 * 64	1	0.89	0.5	0.22	2 * 2 * 64	1 * 1 * 64	1	0.89	0.5	0.44
128C3	4 * 4 * 64	2 * 2 * 128	1	0.89	1.0	0.5	3 * 3 * 64	1 * 1 * 128	1	1.0	0.5	0.5	3 * 3 * 64	1 * 1 * 64	2	1.0	1.0	1.0
128C3	4 * 3 * 128	2 * 1 * 128	2	0.67	1.0	0.5	3 * 3 * 128	1 * 1 * 128	2	1.0	1.0	0.5	3 * 3 * 128	1 * 1 * 32	4	1.0	1.0	1.0
2P2	4 * 2 * 128	2 * 1 * 128	1	0.89	0.5	0.22	2 * 2 * 128	1 * 1 * 128	1	0.89	0.5	0.44	2 * 2 * 128	1 * 1 * 64	2	0.89	1.0	0.89
256C3	3 * 3 * 128	1 * 1 * 256	1	1.0	0.5	0.5	3 * 3 * 128	1 * 1 * 128	2	1.0	1.0	1.0	3 * 3 * 128	1 * 1 * 32	4	1.0	1.0	1.0
256C3	3 * 3 * 256	1 * 1 * 256	2	1.0	1.0	1.0	3 * 3 * 256	1 * 1 * 64	4	1.0	1.0	1.0	3 * 3 * 256	1 * 1 * 16	8	1.0	1.0	1.0
2P2	2 * 2 * 256	1 * 1 * 256	1	0.89	0.5	0.44	2 * 2 * 256	1 * 1 * 128	2	0.89	1.0	0.89	2 * 2 * 256	1 * 1 * 32	4	0.89	1.0	1.0
512C3	3 * 3 * 256	1 * 1 * 256	2	1.0	1.0	1.0	3 * 3 * 256	1 * 1 * 64	4	1.0	1.0	1.0	3 * 3 * 256	1 * 1 * 16	8	1.0	1.0	1.0
512C3	3 * 3 * 512	1 * 1 * 128	4	1.0	1.0	1.0	3 * 3 * 512	1 * 1 * 32	8	1.0	1.0	1.0	3 * 3 * 512	1 * 1 * 8	16	1.0	1.0	1.0
10FC	1 * 1 * 512	1 * 1 * 10	1	0.44	0.02	0.01	1 * 1 * 512	1 * 1 * 10	1	0.89	0.04	0.03	1 * 1 * 512	1 * 1 * 10	2	0.89	0.16	0.14

**Figure 10:**
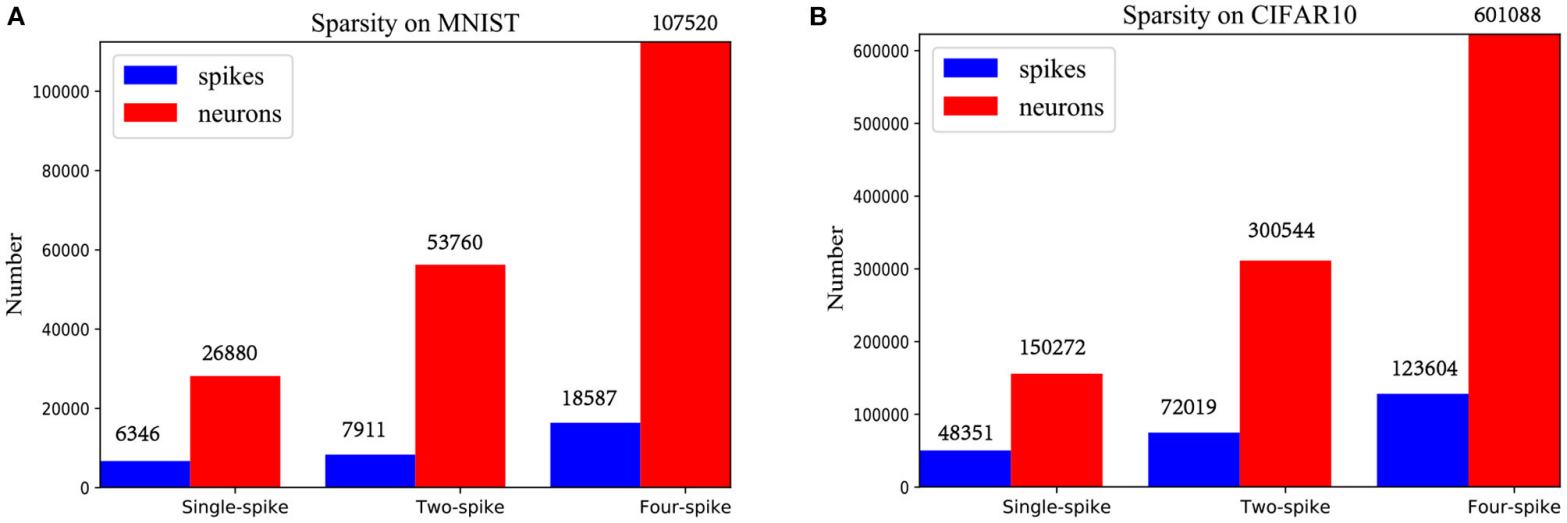
Spiking sparsity of spiking LeNet **(A)** and VGG-Net **(B)** with different precisions.

Moreover, total component neurons and spiking sparsity of LeNet and VGG-Net running on chip are listed in Figure 10. Higher spike precisions significantly bring more spikes and neuron occupations. However, spiking sparsity (spiking times per neuron) is gradually decreasing, from about 0.23 to 0.17 for LeNet and 0.32 to 0.21 for VGG-Net. This result corresponds to the fact that neurons with higher thresholds in an IF neuron group Figure 2 is more difficult to generate spikes.

### 4.4. Speed and Power Analysis

Since this kind of multi-chip system is quite difficult to be instrumented to measure total power, such testing tools are presently undergoing development. For total performance evaluation including network inference speed and power consumption with various model workloads, we adopt a mixed software–hardware methodology as in (Esser et al., 2016; Deng et al., 2020). We run an 8 * 8 chip array in software simulation environment while refer to a actual single-chip performance. For a convolutional or pooling layer less than the capacity of 8 * 8 multi-chip system, such as LeNet, our system can achieve fully unfolded running of this layer within only one computing period. If the size of a layer exceeds the system capacity such as VGG-Net, the direct-memory-access (DMA) controller needs to take data from off-chip memory and write it to the other on-chip SRAM and perform a ping-pong simulation. As provided in Kuang et al. (2021), our chip is operated at a power-supply voltage of 0.9 V, 252 MHz, and achieves up to 21.5 GSOPs and 0.57 pJ/SOP computational performances (idle power contributions are included). The inference latency of each layer of spiking LeNet and VGG-Net for different spike precisions is summarized in Tables 9, 10 It can be seen that all inference latency is at millisecond level but is not linearly proportional to the resource budgets (cores). This is because different spike precisions or model sizes bring different resource utilization as discussed in the last section.

**Table 9:** Chip utilization and latency for LeNet.

	**Single-spike**		**Two-spike**		**Four-spike**	
	**Cores**	**Latency (ms)**	**Cores**	**Latency (ms)**	**Cores**	**Latency (ms)**
16C5	36	0.0549	72	0.1097	144	0.1646
2P2	9	0.0549	16	0.0549	36	0.0549
16C5	4	0.0549	16	0.0549	32	0.0549
2P2	2	0.0549	4	0.0549	8	0.0549
256FC	1	0.0549	1	0.0549	1	0.0549
Total	52	0.2745	109	0.3293	221	0.3842

**Table 10:** Chip utilization and latency for VGG-Net.

	**Single-spike**		**Two-spike**		**Four-spike**	
	**Cores**	**Latency (ms)**	**Cores**	**Latency (ms)**	**Cores**	**Latency (ms)**
64C3	144	0.1646	288	0.2743	576	0.4937
64C3	144	0.1646	288	0.2743	576	0.4937
2P2	36	0.0549	72	0.1097	144	0.1646
128C3	36	0.0549	144	0.1646	288	0.2743
128C3	72	0.1097	144	0.1646	576	0.4937
2P2	18	0.0549	36	0.0549	72	0.1097
256C3	36	0.0549	72	0.1097	288	0.2743
256C3	36	0.0549	144	0.1646	576	0.4937
2P2	9	0.0549	18	0.0549	72	0.1097
512C3	18	0.0549	72	0.1097	288	0.2743
512C3	4	0.0549	16	0.0549	64	0.0549
Total	553	0.8781	1294	1.5362	3520	3.2366

**Figure 11:**
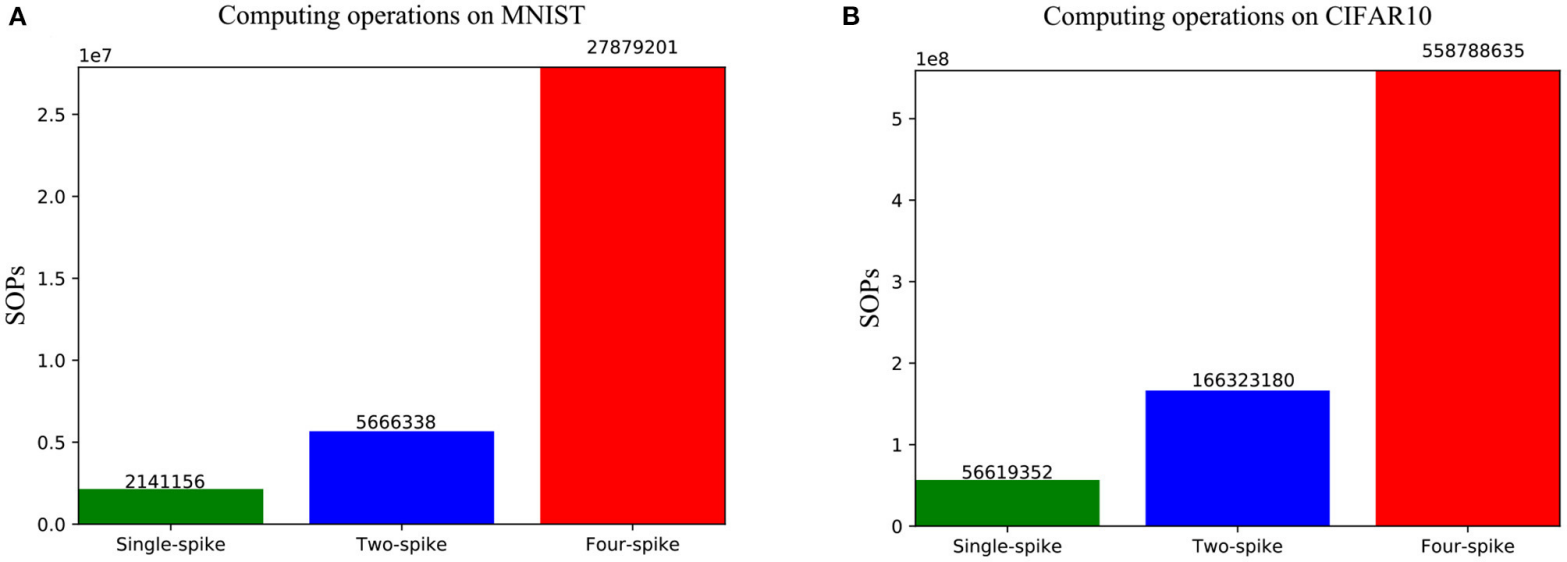
The average number of synaptic operations (SOPs) of one input sample when running spiking LeNet **(A)** and VGG-Net **(B)** with different precisions.

Furthermore, we count the average number of synaptic operations (SOPs) of one sample simulation for spiking LeNet on MNIST and VGG-Net on CIFAR-10 with different spike precisions (see Figure 11). Each synaptic operation delivers a 1-bit spike event through a unique 1-bit non-zero synapse and adds it to membrane potential. It should be noted that for SNNs on our neuromorphic system, no multiplication operation is performed and only low-bit addition is required. Moreover, there are no computation budgets for a synaptic node without spike input, a neuron need update its state only when a spike from the previous layer is coming, so the active power would be proportional to firing activity, i.e., the number of synaptic operations. Total power consumption *P*_*total*_ is the sum of (a) leakage power *P*_*leak*_, which is scaled by measuring idle power for single chip, and (b) active power *P*_*active*_, which can be calculated with the number of SOPs during network inference.

In all cases, the first (the transduction layer) and last layer (the classification layer) are computed off-chip to convert multivalued image inputs into a series of binary spike trains and obtain the final decoding output, respectively. Table 11 shows our results for the evaluated spiking LeNet and VGG-Net on MNIST and CIFAR-10 dataset, with their corresponding accuracies, throughput, power and classifications per energy (FPS per Watt). It can be seen that higher spike precisions for both LeNet and VGG-Net bring higher classification accuracy but larger inference power and latency. The four-spike LeNet and VGG-Net on chips achieve a real-time inference speed of 0.38 ms/image, 3.24 ms/image, and an average power consumption of 0.28 and 2.3 mJ/image, respectively, at 0.9 V, 252 MHz. Compared with GPUs (Titan Xp and Tesla V100) computing with the default FP32 precision, our system can obtain comparable accuracies but nearly two orders of magnitude power efficiency improvements. On the other hand, our results show that we can achieve a close classification speed on CIFAR-10 compared with TrueNorth (Esser et al., 2016) and even faster than Tianjic chip (Deng et al., 2020). The weakness in power efficiency (FPS/W) results from heavy communication workloads for off-chip memory access and inter-chip routing because of the relatively smaller (8 * 8) system capacity for ours, while the other two adopt quite large-scale multi-core design (4,096 for TrueNorth, 156 for Tianjic) and asynchronous communication protocol (TrueNorth).

**Table 11:** Summary of main performance.

	**Models**	**Accuracy**	**FPS^*a*^**	**mJ^*b*^**	**FPS/W**
MNIST	Single-spike (**This work**)	99.27%	3642	0.1897	5271
	Two-spike (**This work**)	99.32%	3036	0.2293	4361
	Four-spike (**This work**)	99.37%	2602	0.2751	3635
	TrueNorth (Esser et al., 2015)	99.42%	1000	0.121	8264
	Tianjic (Deng et al., 2020)	99.48%	2126	0.069	14555
	Titan Xp (FP32 precision)	99.52%	1433	35	29
	V100 (FP32 precision)	99.52%	2185	22	45
CIFAR-10	Single-spike (**This work**)	89.12%	1138	0.6148	1626
	Two-spike (**This work**)	90.95%	650	1.0854	921
	Four-spike (**This work**)	91.91%	308	2.3013	434
	TrueNorth (Esser et al., 2016)	83.41%	1249	0.1637	6109
	Tianjic (Deng et al., 2020)	93.52%	1751	0.12	8217
	Titan Xp (FP32 precision)	92.85%	617	67	15
	V100 (FP32 precision)	92.85%	1181	42	24

a *FPS is denoted as frames/second and FPS/W is fames/second per Watt*.

b *The average energy consumption for one input frame inference*.

## 5. Conclusion and Discussion

In this work, we introduce an adjustable quantization and training algorithm for ANNs to minimize common spike approximation errors, and propose a scatter-and-gather rate-based conversion method for SNNs built with simple IF neurons. Besides, we develop an incremental and resource-efficient mapping framework for these SNNs on a reconfigurable neuromorphic ASIC. Experimental results show that our spiking LeNet on MNIST and VGG-Net on CIFAR-10/100 dataset yield great classification accuracies. Meanwhile, the employment with our presented mapping algorithm is able to flexibly manage network topology placement on target neuromorphic chip with maximum resource efficiency. The four-spike LeNet on MNIST and VGG-Net CIFAR-10 on our system achieve millisecond-level speed and millijoule-level power. It should be noted that in this power and speed evaluation stage, we treat the inter-chip communication identical with the intra-chip one. However, for a normal multi-chip system, the inter-chip communication is usually more expensive. Hence, integrating multiple computing cores into a single chip to reduce inter-chip communication is a main future work. Besides, a more thoughtful mapping scheme with the consideration of overall resource and communication can also help to alleviate cross-chip overhead. For more complicated applications, future works will concentrate on the conversion and mapping function on other architecture such as ResNet and RNN. A more rewarding work is to try training and mapping of hybrid-precision models. This may bring a further performance improvement on this neuromorphic chip.

## Data Availability Statement

The original contributions presented in the study are included in the article/supplementary material, further inquiries can be directed to the corresponding author/s.

## Author Contributions

CZ and XC proposed the idea, designed, and conducted the experiments. YK and KL helped to complete hardware measurement and software simulation. CZ, XC, and YW wrote the manuscript, then RH revised it. XC, YW, and XW directed the project and provided overall guidance. All authors contributed to the article and approved the submitted version.

## Funding

This work was supported by the National Key Research and Development Program of China (Grant No. 2018YFB2202605), National Natural Science Foundation of China (Grant No. 61421005), the 111 Project (B18001), and R&D Project of Shenzhen Science and Technology Innovation Committee (Grant Nos. JCYJ20200109120404043 and KQTD20200820113105004).

## Conflict of Interest

The authors declare that the research was conducted in the absence of any commercial or financial relationships that could be construed as a potential conflict of interest.

## Publisher's Note

All claims expressed in this article are solely those of the authors and do not necessarily represent those of their affiliated organizations, or those of the publisher, the editors and the reviewers. Any product that may be evaluated in this article, or claim that may be made by its manufacturer, is not guaranteed or endorsed by the publisher.

## Footnotes

^1^ https://github.com/edwardzcl/Spatio_temporal_SNNs

^2^In this paper, we focus on neural networks with batch normalization, but our quantization method can also support other architectures without that.

^3^*mCn* represents a convolutional layer with *m* filters and filter size of *n* * *n*. *mPn* is a pooling layer with *m* * *m* size and stride of *n*. It should be noted that we use convolution with stride of 2 to replace pooling. mFC is the fully connected layer with *m* neurons.

^4^ http://www.cs.toronto.edu/~kriz/cifar.html
